# Supplementary material for: Rapid Screening and Comparison of Chimeric Lysins for Antibacterial Activity against Staphylococcus aureus Strains
Source: Antibiotics (Basel). 2023 Mar 29;12(4):667. doi: 10.3390/antibiotics12040667 (PMC10135017; doi:10.3390/antibiotics12040667)
Supplement: Supplementary file 1 [file antibiotics-12-00667-s001.zip › antibiotics-2274971-supplementary.pdf]

antibiotics
MDPI

Supplementary Information

Rapid Screening and Comparison of Chimeric Lysins for Antibacterial Activity against *Staphylococcus aureus* Strains (Supplementary Information)

Jin-Mi Park [1,2], Dae-Sung Ko [1,2], Hee-Soo Kim [1,2], Nam-Hyung Kim [1,2], Eun-Kyoung Kim [3], Young-Hye Roh [3], Danil Kim [3], Jae-Hong Kim [1], Kang-Seuk Choi [2,4,]* and Hyuk-Joon Kwon [1,2,]*

1 Laboratory of Poultry Medicine, College of Veterinary Medicine, Seoul National University, Seoul 08826, Republic of Korea
2 Research Institute for Veterinary Science, College of Veterinary Medicine, BK21 for Veterinary Science, Seoul 08826, Republic of Korea
3 Department of Farm Animal Medicine, College of Veterinary Medicine, Seoul National University, Pyeongchang-gu 25354, Republic of Korea
4 Laboratory of Avian Diseases, College of Veterinary Medicine, Seoul National University, Seoul 08826, Republic of Korea
* Correspondence: kchoi0608@snu.ac.kr (K.-S.C.); kwonhj01@snu.ac.kr (H.-J.K.); Tel.: +82-2-880-1266 (K.-S.C. & H.-J.K.)

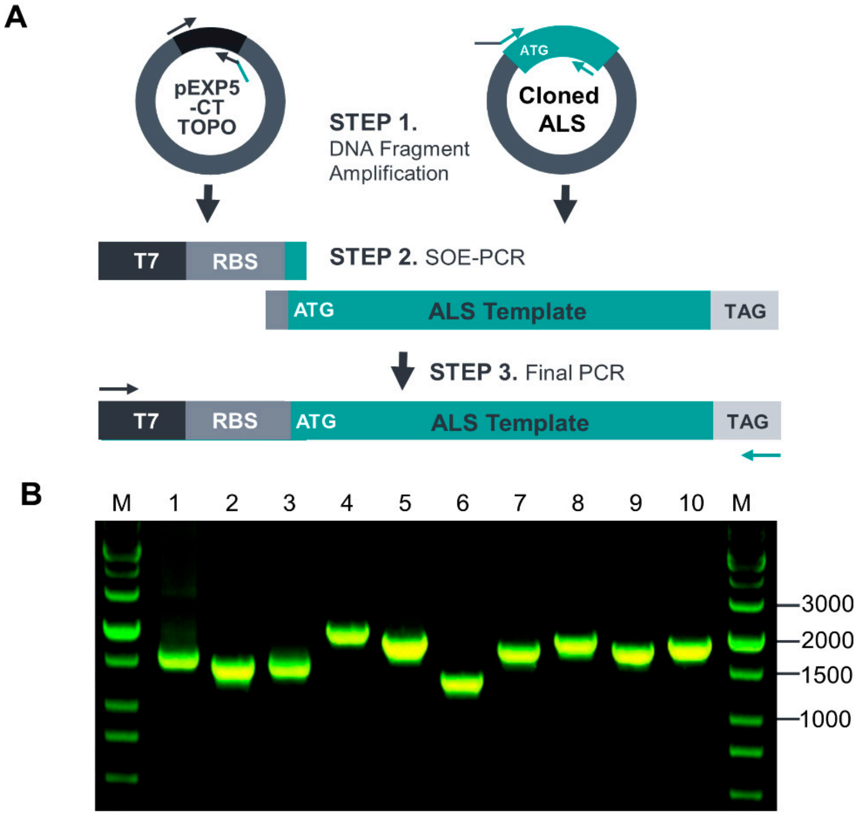

Figure S1. Preaparation of chimeric lysin DNA template for cell-free expression system by PCR and SOE-PCR. (A) Schematic presentation of SOE-PCR for DNA template preparation and (B) Agarose gel electroporation of high-concentration purified DNA template for cell-free expression. Samples (about 400-600 ng/μl) were loaded with 1 μl each on 1.3% agarose gel. Lane M, 1000 bp size marker; Lane 1, ALS1 (437ng), 1576bp; Lane 2, ALS2 (548ng), 1429bp; Lane 3, ALS3 (423ng), 1435bp; Lane 4,

ALS4 (483ng), 2032bp; Lane 5, ALS7 (600ng), 1858bp; Lane 6, ALS6 (403ng), 1330bp; Lane 7, ALS7 (420ng), 1671bp; Lane 8, ALS8 (417ng), 1936bp; Lane 9, ALS9 (514ng), 1783bp; Lane 10, ALS10 (518ng), 1879bp.

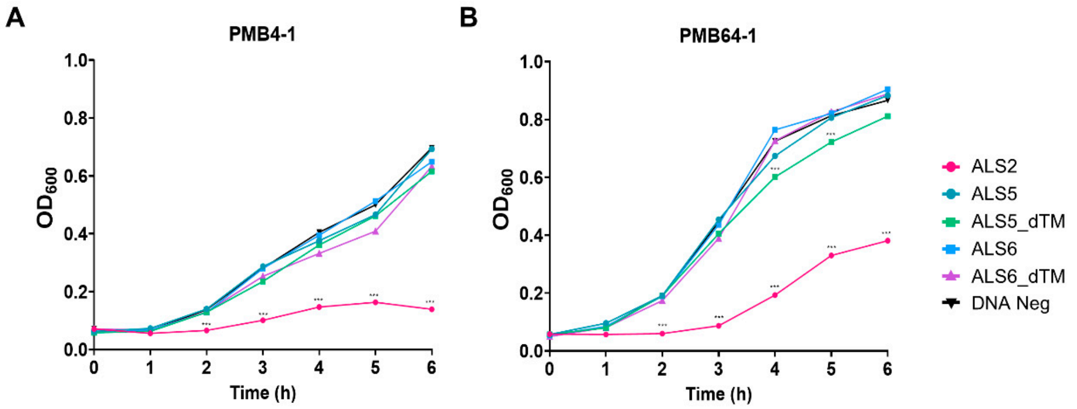

Figure S2. Effect of transmembrane peptides on antibacterial activity of ALS5 and ALS6. Antibacterial activity of parents (ALS5 and ALS6) and mutants (ALS5-dTM and ALS6-dTM) without transmembrane peptides were prepared using cell-free expression system and antibacterial activity was tested using turbidity reduction test. Antibacterial activity of chiemric lysins to *S. aureus* strains (A) PMB4-1 and (B) PMB64-1. ALS2 and cell free expression reaction without DNA were used as positive and negative controls. Single experiment was performed in triplicate samples. Data were analyzed by one-way ANOVA followed by Dunnett's test to determine the significance relative to the negative control. (p *** < 0.001, p ** < 0.01, p * < 0.05).

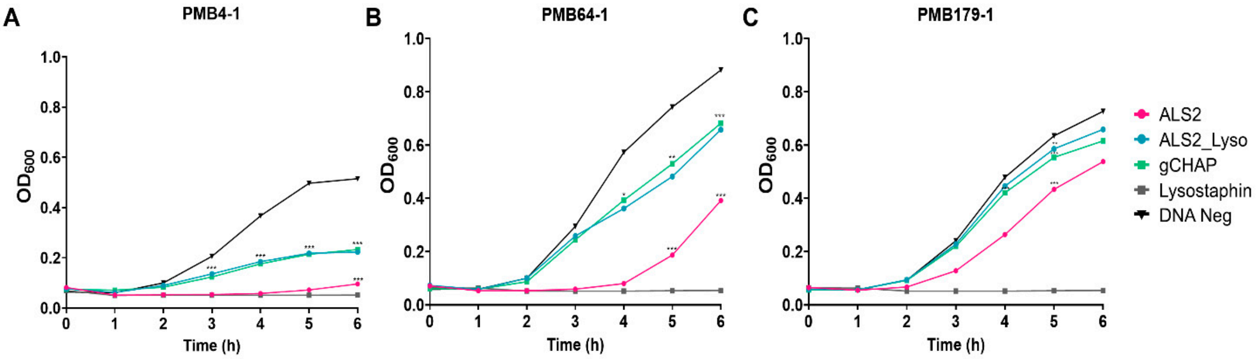

Figure S3. Comparison of antibacterial activities of ALS2, ALS2_Lyso and gCHAP. Antibacterial activity of chiemric lysins to *S. aureus* strains (A) PMB4-1, (B) PMB64-1 and (C)PMB179-1. Lysostaphin and cell free expression reaction without DNA were used as positive and negative controls. Single experiment was performed in triplicate samples. Data were analyzed by one-way ANOVA followed by Dunnett's test to determine the significance relative to the negative control. (p *** < 0.001, p ** < 0.01, p * < 0.05).

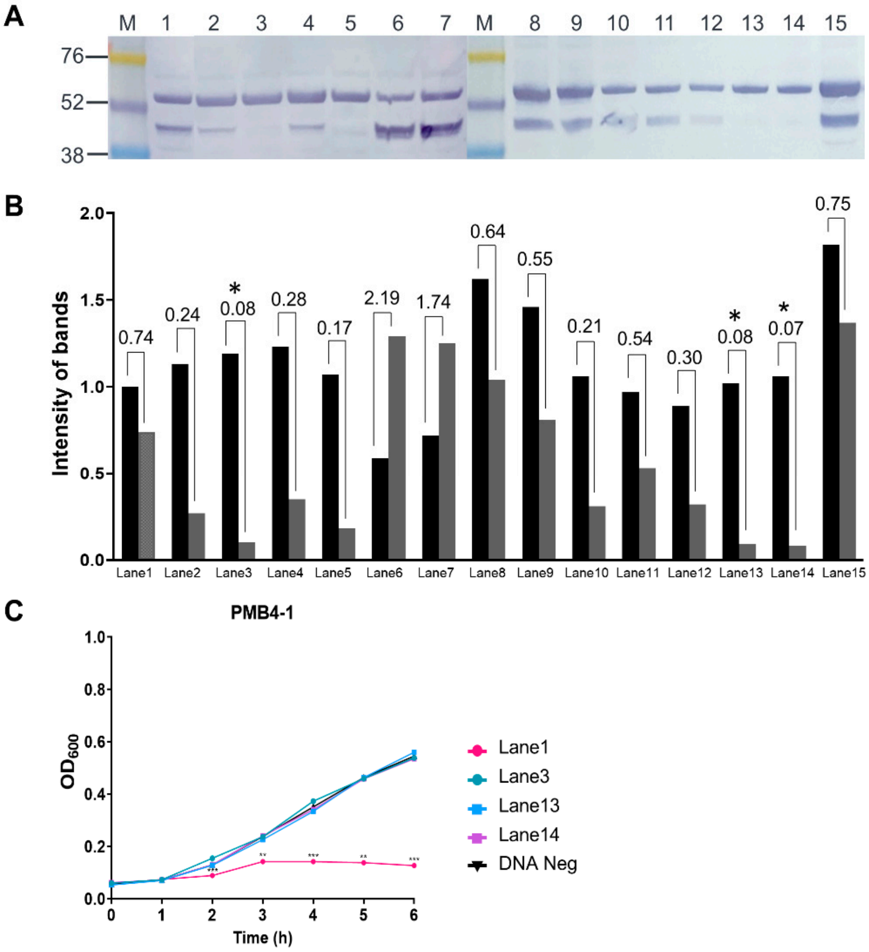

Figure S4. Effects of 89th methionine mutations on expression of the subprotein and antibacterial activity. (A) Western analysis in ALS2 89th amino acid (methionine) mutation. Lane M: protein molecular weight marker (Amersham™ ECL™ Rainbow™ Marker, Lane 1: ALS2 ; Lane 2: ATC (Ile) mutagenesis ; Lane 3: CTG (Leu) mutagenesis ; Lane 4: TTG (Leu) mutagenesis ; Lane 5: GTG (Val) mutagenesis ; Lane 6: ATT (Ile) mutagenesis ; Lane 7: ATA (Ile) mutagenesis ; Lane 8: TGT (Cys) mutagenesis ; Lane 9: TGC (Cys) mutagenesis ; Lane 10: ACT (Thr) mutagenesis ; Lane 11: ACC (Thr) mutagenesis ; Lane 12: ACA (Thr) mutagenesis ; Lane 13: ACG (Thr) mutagenesis ; Lane 14: GGG (Gly) mutagenesis ; Lane 15: GGC (Gly) mutagenesis. (B) The relative protein intensity of ALS2 protein and subprotein according to mutagenesis. Protein expression ratio was presented using Image J compared to ALS2 (52.4kDa). (C) Antibacterial activity of CTG (Leu), ACG (Thr), GGG (Gly) mutants were compared with parent ALS2. Cell free expression reaction without DNA was used as negative controls. Single experiment was performed in triplicate samples. Data were analyzed by one-way ANOVA followed by Dunnett's test to determine the significance relative to the negative control. (p *** < 0.001, p ** < 0.01, p * < 0.05).

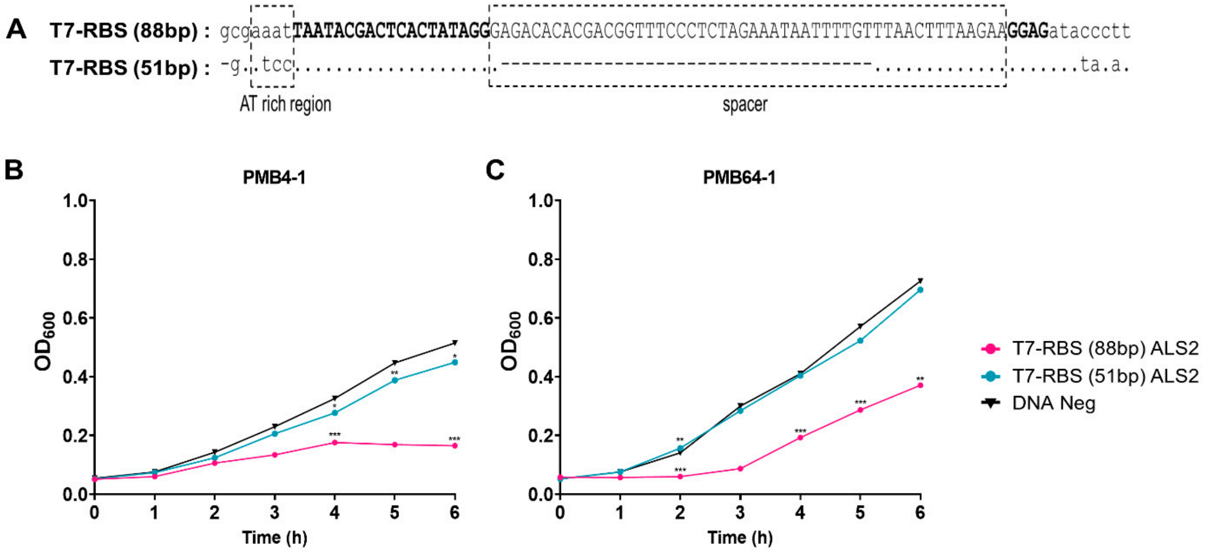

Figure S5. Comparison of the present and previous cell-free expression systems. (A) Structure of the present (88bp) and the previous (51bp) T7 promoter and ribosome-binding site (RBS). AT-rich and spacer regions are boxed in broken line and T7 promoter and RBS sequences are in bold. Comparison of antibacterial activities of ALS2 prepared by the present and previous methods in *S. aureus* strains (B) PMB4-1 and (C) PMB64-1. Cell free expression reaction without DNA was used as negative controls. Single experiment was performed in triplicate samples. Data were analyzed by one-way ANOVA followed by Dunnett's test to determine the significance relative to the negative control. (p *** < 0.001, p ** < 0.01, p * < 0.05).

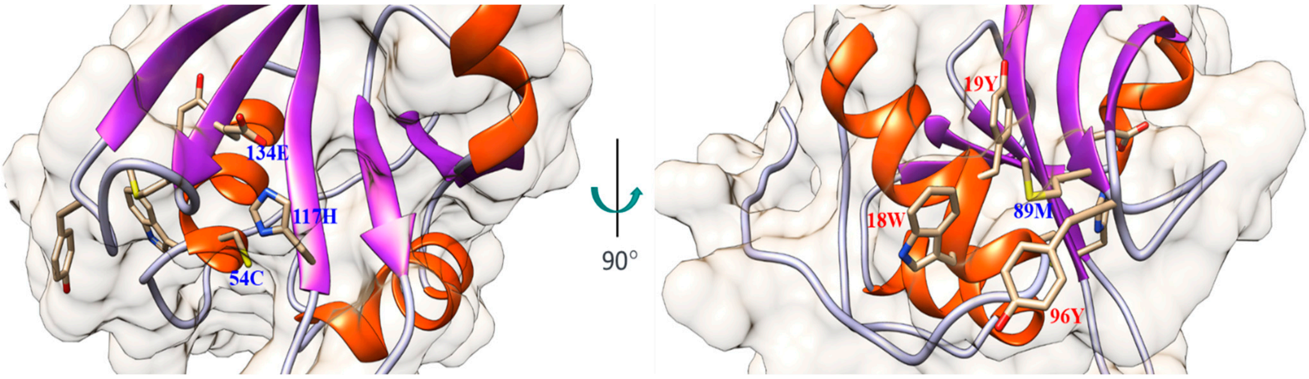

(**A**) (**B**)

Figure S6. Locations of catalytic triad and 89M side chain in the 3D structure of gCHAP (AF-A0A6B5FVD2-F1-model_v3). (A) Location of catlytic triad (54C, 117H, and 134E). (B) Location of 89M side chain and interaction with neighboring side chains of 18W, 19Y and 96Y.

Table S1. Proteins and domains used for chimeric lysin construction.

Abbreviated name	Genbank Protein Full name	Genbank accession No.	Amino acid Region	Amino acid Length
**ØK**	LysK	AFN38929	1-495	495
**gCHAP**	Secretory antigen SsaA-like protein	ABD29804	152-265 (Surface antigen)	114
**gAmi**	Autolysin	CYF13637	257-385 (Ami_2 domain)	129
**LytM**	glycine-glycine endopeptidase LytM	WP_000736798	1-316	316
**LytH**	Cell wall amidase lyth	QBX59665	1-291	291
**LytD**	N-acetylglucosaminidase	WP_000739230	1-258	258
**SsaA**	Secretory antigen SsaA-like protein	ABD29804	1-265	265
**Lysostaphin**[a]	lysostaphin (ttg start codon)	AAA26655.1	144-389	246

[a] used as a positive sample in the screening test.

Table S2. PCR primers used for amplification of lysin, autolysin and lysostaphin

Amplified gene	Primer name	Sequence (5' -3')	Usage
LysK	phiK CHAP-F	ATGGCAAAGACTCAGGCAGA	ALS1,3 amplification
	phiK CHAP-R	TTCCTTCTTAACAGTAGTACC	ALS3 amplification
	phiK Ami-F	GCAGGTACTACTGTTAAGAAGG	ALS2,4,5,10 amplification
	phiK SH3-F	GGTACTTCTTCTTCTACTGTC	ALS3 amplification
	phiKL-R	TTTGAAAACACCCCATGCAAC	ALS1,2,3,4,5,10 & 5-dTM amplification
LysM peptidoglycan-binding domain-containig protein	gCHAP-F	atgGCATCATCTTTTAATCACCAA	ALS2 amplification
	gCHAP-R	ATGGATGAATGCATAGCTAGA	ALS2,6,9,10 & 6-dTM amplification
Autolysin (AtlE)	gAmi2-F	CCTAAATACGCATACCGTAAC	ALS3 amplification
	gAmi2-R	GTCATATAATTGATCATAACT	ALS3 amplification
Glycine-glycine endopeptidase LytM	LytM-F	ATGAAAAAATTAACAGCAGCAGC	ALS4,8 amplification
	LytM-R	TCTACTTTGCAAGTATGACGTTGG	ALS4,8 amplification
Cell wall amidase lyth	LytH-F	ATGAAAAAAATAGAGGCATGGTT	ALS6,7,8,9 amplification
	LytH-R	CGCAGAAAAATAAATTTTAAG	ALS6,7,8,9 amplification
N-acetylglucosaminidase	LytD-F	ATGAAGAAGAATTTCAAGTTACG	ALS5,7 amplification
	LytD-R	TTTATAATATGTTTGTCTAAT	ALS5,7 amplification
Secretory antigen SsaA-like protein	SsaA-F	aTGAAAAAATTAGCATTTGCAATA	ALS9,10 amplification

Lysostaphin (ttg start codon)	Lyso-F	atgGCGGCAACGCATGAACAC	Lysostaphin amplification
	Lyso-R	TTTGATAGTACCCCACAGAAC	Lysostaphin amplification

small case letters indicate nucleotides added artificially.
Underlined letters indicate the sequence of T7 and RBS fragments.

Table S3. PCR primers used for amplification of DNA templates for cell free expression

Primer name	Sequence (5' -3')	Usage
T7-F	GCGAAATTAATACGACTCACTATAG	T7 amplification PCR for all
T7-R	AAGGGTAT**CTCC**TTCTTAAAGTTAAAC	T7 amplification
phiKL-TAG-R	ctaTTTGAAAACACCCCATGCAAC	ALS1,2,3,4,5,10 & 5-dTM amplification
gCHAP-TAG-R	ctaATGGATGAATGCATAGCTAGA	ALS6 & 6-dTM amplification
LytD-TAG-R	ctaTTTATAATATGTTTGTCTAAT	ALS7 amplification
LytM-TAG-R	ctaTCTACTTTGCAAGTATGACGTTGG	ALS8 amplification
LytH-TAG-R	ctaCGCAGAAAAATAAATTTTAAG	ALS9 amplification
Lyso-TAG-R	ctaTTTGATAGTACCCCACAGAAC	Lysostaphin amplification
T7-phiKCHAP-F	CTTTAAGAA**GGAG**ATACCCTTATGGCAAA-GACTCAGGCAG	SOE-PCR for ALS1, ALS3
T7-phiKCHAP-R	CTGCCTGAGTCTTTGCCATAAGGG-TAT**CTCC**TTCTTAAAG	SOE-PCR for ALS1, ALS3
T7-gCHAP-F	CTTTAAGAA**GGAG**ATACCCTTATGG-CATCATCTTTTAATCAC	SOE-PCR for ALS2
T7-gCHAP-R	GTGATTAAAAGATGATGCCATAAGGG-TAT**CTCC**TTCTTAAAG	SOE-PCR for ALS2
T7-LytM-F	CTTTAAGAA**GGAG**A-TACCCTTATGAAAAAATTAACAGCAGC	SOE-PCR for ALS4
T7-LytM-R	GCTGCTGTTAATTTTTTCATAAGGG-TAT**CTCC**TTCTTAAAG	SOE-PCR for ALS4
T7-LytD-F	CTTTAAGAA**GGAG**ATACCCTTATGAAGAA-GAATTTCAAGTTAC	SOE-PCR for ALS5
T7-LytD-R	GTAACTTGAAATTCTTCTTCATAAGGG-TAT**CTCC**TTCTTAAAG	SOE-PCR for ALS5
T7-LytH-F	CTTTAAGAA**GGAG**A-TACCCTTATGAAAAAAATAGAGGCATG	SOE-PCR for ALS6,7,8
T7-LytH-R	CATGCCTCTATTTTTTTCATAAGGG-TAT**CTCC**TTCTTAAAG	SOE-PCR for ALS6,7,8
T7-SsaA-F	CTTTAAGAA**GGAG**A-TACCCTTATGAAAAAATTAGCATTTGC	SOE-PCR for ALS9,10
T7-SsaA-R	GCAAATGCTAATTTTTTCATAAGGG-TAT**CTCC**TTCTTAAAG	SOE-PCR for ALS9,10
T7-Lyso-F	CTTTAAGAAGGAGATACCCTTATGGCGG-CAACGCATGAAC	SOE-PCR for Lysostaphin
T7-Lyso-R	GTTCATGCGTTGCCGCCATAAGGG-TATCTCCTTCTTAAAG	SOE-PCR for Lysostaphin

small case letters indicate nucleotides added artificially.
Underlined letters indicate the sequence of T7 and RBS fragments.

Table S4. PCR primers used for ALS2 mutagenesis.

Primer name	Location	Modified sequence (5′-3′)	Usage
GAT81GAC-F	229-255	CGTGTCAATGGTGACGGTAGTATCTTG	TS-10 mutation
GAT81GAC-R	229-255	CAAGATACTACCGTCACCATTGACACG	TS-10 mutation
GCT73GCC-F	203-234	CATATGGTCATGTTGCCTACGTTGAACGTGTC	TS-34 mutation
GCT73GCC-R	203-234	GACACGTTCAACGTAGGCAACATGACCATATG	TS-34 mutation
TAT74TAC-F	208-238	GGTCATGTTGCCTACGTTGAACGTGTCAATG	TS-31 mutation
TAT74TAC-R	208-238	CATTGACACGTTCAACGTAGGCAACATGACC	TS-31 mutation
GAA88TAA-F	245-277	GTAGTATCTTGATTTCTTAAATGAATTACACAT	STOP mutation
GAA88TAA-R	245-277	ATGTGTAATTCATTTAAGAAATCAAGATACTAC	STOP mutation

Table S5. PCR primers used for transmembrane region deletion.

Primer name	Sequence (5' -3')	Usage
ALS5-dTM_F	atgGTGAATGAAACTAAATTGTTTAAAAAT	ALS5-dTM amplification
ALS6-dTM_F	atgAATAGCAATAGTGAAGATAGTGGGAAC	ALS6-dTM amplification
T7-ALS5-dTM_F	CTTTAAGAAGGAGA-<br>TACCCTTATGGTGAATGAAACTAAATT-<br>GTTTAAAAAT	SOE-PCR for ALS5-dTM
T7-ALS6-dTM_F	CTTTAAGAAGGAGATACCCTTATGAA-<br>TAGCAATAGTGAAGATAGTGGGAAC	SOE-PCR for ALS6-dTM

Table S6. PCR primers used for mutagenesis of ALS2 89 th amino acid (methionine).

Primer name	Modified sequence (5′-3′)	Amino acid change
gCHAP-ATG89ATC-F	CTTGATTTCTGAAATCAATTACACATATGGTCC	Isoleucine
gCHAP-ATG89ATC-R	GGACCATATGTGTAATTGATTTCAGAAATCAAG	Isoleucine
gCHAP-ATG89CTG-F	GTATCTTGATTTCTGAACTGAATTACACATATGGTCC	leucine
gCHAP-ATG89CTG-R	GGACCATATGTGTAATTCAGTTCAGAAATCAAGATAC	leucine
gCHAP-ATG89TTG-F	GTATCTTGATTTCTGAATTGAATTACACATATGGTC	leucine
gCHAP-ATG89TTG-R	GACCATATGTGTAATTCAATTCAGAAATCAAGATAC	leucine
gCHAP-ATG89GTG-F	GTATCTTGATTTCTGAAGTGAATTACACATATGGTC	valine
gCHAP-ATG89GTG-R	GACCATATGTGTAATTCACTTCAGAAATCAAGATAC	valine
gCHAP-ATG89ATT-F	CTTGATTTCTGAAATTAATTACACATATGGTCC	Isoleucine
gCHAP-ATG89ATT-R	GGACCATATGTGTAATTAATTTCAGAAATCAAG	Isoleucine
gCHAP-ATG89ATA-F	CTTGATTTCTGAAATAAATTACACATATGGTCC	Isoleucine
gCHAP-ATG89ATA-R	GGACCATATGTGTAATTTATTTCAGAAATCAAG	Isoleucine
gCHAP-ATG89TGT-F	GTATCTTGATTTCTGAATGTAATTACACATATGGTCC	cysteine
gCHAP-ATG89TGT-R	GGACCATATGTGTAATTACATTCAGAAATCAAGATAC	cysteine
gCHAP-ATG89TGC-F	GTATCTTGATTTCTGAATGCAATTACACATATGGTCC	cysteine
gCHAP-ATG89TGC-R	GGACCATATGTGTAATTGCATTCAGAAATCAAGATA	cysteine
gCHAP-ATG89ACT-F	GTATCTTGATTTCTGAAACTAATTACACATATGGTCC	threonine
gCHAP-ATG89ACT-R	GGACCATATGTGTAATTAGTTTCAGAAATCAAGATAC	threonine

gCHAP-ATG89ACC-F	GTATCTTGATTTCTGAAACCAATTACACATATGGTCC	threonine
gCHAP-ATG89ACC-R	GGACCATATGTGTAATTGGTTTCAGAAATCAAGATAC	threonine
gCHAP-ATG89ACA-F	GTATCTTGATTTCTGAAACAAATTACACATATGGTCC	threonine
gCHAP-ATG89ACA-R	GGACCATATGTGTAATT TGTTTCAGAAATCAAGATAC	threonine
gCHAP-ATG89ACG-F	GTATCTTGATTTCTGAAACGAATTACACATATGGTCC	threonine
gCHAP-ATG89ACG-R	GGACCATATGTGTAATTCGTTTCAGAAATCAAGATAC	threonine
gCHAP-ATG89GGG-F	GTATCTTGATTTCTGAAGGGAATTACACATATGGTCC	glycine
gCHAP-ATG89GGG-R	GGACCATATGTGTAATTCCCTTCAGAAATCAAGATAC	glycine
gCHAP-ATG89GGC-F	GTATCTTGATTTCTGAAGGCAATTACACATATGGTCC	glycine
gCHAP-ATG89GGC-R	GGACCATATGTGTAATTGCCTTCAGAAATCAAGATAC	glycine

Underlined letters indicate nucleotides modified by mutagenesis.
